# Spinal Mobilization Prevents NGF-Induced Trunk Mechanical Hyperalgesia and Attenuates Expression of CGRP

**DOI:** 10.3389/fnins.2020.00385

**Published:** 2020-04-30

**Authors:** William R. Reed, Joshua W. Little, Carla R. Lima, Robert E. Sorge, Ceren Yarar-Fisher, Mualla Eraslan, Christopher P. Hurt, Timothy J. Ness, Jianguo G. Gu, Daniel F. Martins, Peng Li

**Affiliations:** ^1^Department of Physical Therapy, University of Alabama at Birmingham, Birmingham, AL, United States; ^2^Rehabilitation Sciences Program, University of Alabama at Birmingham, Birmingham, AL, United States; ^3^Department of Surgery, Center for Anatomical Science and Education, Saint Louis University School of Medicine, St. Louis, MO, United States; ^4^Department of Psychology, University of Alabama at Birmingham, Birmingham, AL, United States; ^5^Department of Physical Medicine and Rehabilitation, University of Alabama at Birmingham, Birmingham, AL, United States; ^6^Department of Anesthesiology and Perioperative Medicine, University of Alabama at Birmingham, Birmingham, AL, United States; ^7^Postgraduate Program in Health Sciences, Experimental Neuroscience Laboratory (LaNEx), University of Southern Santa Catarina, Palhoça, Brazil; ^8^School of Nursing, University of Alabama at Birmingham, Birmingham, AL, United States

**Keywords:** spinal mobilization, manual therapy, nerve growth factor, NGF, calcitonin gene-related peptide, CGRP, low back pain, mechanical hyperalgesia

## Abstract

**Introduction:**

Low back pain (LBP) is a complex and growing global health problem in need of more effective pain management strategies. Spinal mobilization (SM) is a non-pharmacological approach recommended by most clinical guidelines for LBP, but greater utilization and treatment optimization are hampered by a lack of mechanistic knowledge underlying its hypoalgesic clinical effects.

**Methods:**

Groups of female Sprague-Dawley rats received unilateral trunk (L5 vertebral level) injections (50 μl) of either vehicle (phosphate-buffer solution, PBS; VEH) or nerve growth factor (NGF; 0.8 μM) on Days 0 and 5 with or without daily L5 SM (VEH, NGF, VEH + SM, VEH + SM). Daily passive SM (10 min) was delivered by a feedback motor (1.2 Hz, 0.9N) from Days 1 to 12. Changes in pain assays were determined for mechanical and thermal reflexive behavior, exploratory behavior (open field events) and spontaneous pain behavior (rat grimace scale). On Day 12, lumbar (L1–L6) dorsal root ganglia (DRG) were harvested bilaterally and calcitonin gene-related peptide (CGRP) positive immunoreactive neurons were quantified from 3 animals (1 DRG tissue section per segmental level) per experimental group.

**Results:**

NGF induced bilateral trunk (left *P* = 0.006, right *P* = 0.001) mechanical hyperalgesia and unilateral hindpaw allodynia (*P* = 0.006) compared to the vehicle group by Day 12. Additionally, we found for the first time that NGF animals demonstrated decreased exploratory behaviors (total distance traveled) and increased grimace scale scoring compared to the VEH group. Passive SM prevented this development of local (trunk) mechanical hyperalgesia and distant (hindpaw) allodynia, and normalized grimace scale scores. NGF increased CGRP positive immunoreactive neurons in ipsilateral lumbar DRGs compared to the VEH group ([L1]*P* = 0.02; [L2]*P* = 0.007) and SM effectively negated this increase in pain-related neuropeptide CGRP expression.

**Conclusion:**

SM prevents the development of local (trunk) NGF-induced mechanical hyperalgesia and distant (hindpaw) allodynia, in part, through attenuation of CGRP expression in lumbar DRG sensory neurons. NGF decreases rat exploratory behavior and increases spontaneous pain for which passive SM acts to mitigate these pain-related behavioral changes. These initial study findings suggest that beginning daily SM soon after injury onset might act to minimize or prevent the development of LBP by reducing production of pain-related neuropeptides.

## Introduction

Low back pain (LBP) is a poorly managed, costly, and rapidly growing health problem that results in more disability than any other condition globally (Dagenais et al., 2008; Hoy et al., 2014; Dieleman et al., 2016). Thus, there exists an urgent need for more clinically effective therapeutic interventions or strategies that can minimize LBP severity and/or prevent the transition from acute to chronic LBP. Mild-to-moderate treatment efficacy, cost effectiveness, and high patient satisfaction (Bronfort et al., 2008; Hurwitz, 2012; Hidalgo et al., 2014; Nahin et al., 2016) have contributed to an increased number of clinical practice guidelines recommending the use of passive spinal manual therapy (spinal manipulation and spinal mobilization) for the treatment of non-specific LBP (Chou et al., 2017). However, a lack of a knowledge regarding physiological mechanisms responsible for spinal manual therapy-induced pain relief, as well as the inability to identify the most appropriate clinical subpopulations most likely to therapeutically benefit have severely hampered spinal manual therapy optimization and increased clinical utilization.

A recent systematic review of spinal mobilization (SM) reported existing evidence for SM-related physiological effects on sympathoexcitation, decreased neural mechanosensitivity, mechanical hypoalgesia, and improved muscle function (Lascurain-Aguirrebena et al., 2016). Mobilization has been reported to induce local and/or distant antihyperalgesic/analgesic effects in both preclinical models (Martins et al., 2012, 2013a,b; Santos et al., 2018; Salgado et al., 2019), and human studies (Vicenzino et al., 1996; La Touche et al., 2013; Salom-Moreno et al., 2014) but mechanistic knowledge related to peripheral/central pain processing remains severely limited. This gap in knowledge of specific anatomical structures, pathways, and molecular mechanisms responsible for SM-induced antihyperalgesia/analgesia is due in large part to a lack of adequate clinically relevant preclinical LBP models and non-reflex oriented behavioral assays in which to investigate LBP mechanisms and biological effects of pharmacological and non-pharmacological (active or passive) therapeutic interventions. LBP models that use minimally invasive techniques and/or endogeneously synthetized molecules may be preferable to some of the more invasive or traditional inflammatory LBP animal models (Shi et al., 2018).

Use of nerve growth factor (NGF) in a LBP preclinical model was first described by Hoheisel et al. (2013). They demonstrated that two unilateral NGF injections (delivered 5 days apart) into the adult male rat lumbar multifidi muscles results in persistent localized (unilateral) low back (trunk) mechanical hyperalgesia or LBP. The onset of this NGF-induced mechanical hyperalgesia started within hours after the second NGF injection and persisted through the end of their experimental period at Day 14. While the precise neurobiological mechanisms by which NGF induces sustained mechanical trunk hyperalgesia are not completely understood, evidence indicates that sensitization of skeletal muscle nociceptors (Mann et al., 2006; Murase et al., 2010), glial-dependent latent sensitization of dorsal horn neurons (Zhang et al., 2017), and spinal dorsal horn neuron hyperexcitability immediately following the 2nd NGF injection (Hoheisel et al., 2007, 2013; de Azambuja et al., 2018) all contribute. NGF is an endogenously produced neurotrophin involved in pain transduction that binds to tyrosine kinase receptor A in nociceptors (Klein et al., 1991; Pezet et al., 1999). It is implicated in the regulation of prolonged mechanical/thermal hyperalgesia in animals and humans (Dyck et al., 1997; Murase et al., 2010; Mills et al., 2013; Eskander et al., 2015; Kras et al., 2015), and when administered systemically produces widespread myalgias in humans, with the greatest frequency of myalgia occurring in the low back (Petty et al., 1994). NGF is released in skeletal muscle and is thought to contribute greatly to delayed onset muscle soreness (Nie et al., 2009) and increases are associated with greater pain behaviors (Murase et al., 2010; Hayashi et al., 2011). Clinical relevancy of NGF in musculoskeletal pain conditions is clearly evidenced by anti-NGF therapies which are currently in phase III clinical trials for the management of LBP (Miller et al., 2017; Norman and McDermott, 2017).

Despite multiple biological mechanisms contributing to the development of NGF-induced trunk mechanical hyperalgesia, increased nociceptive neuropeptide expression is most certainly involved as NGF has been associated with upregulation of calcitonin gene-related peptide (CGRP) in the dorsal root ganglia (DRG) and spinal cord dorsal horn (Malcangio et al., 1997; Supowit et al., 2001; Kimura et al., 2014). Activation of CGRP receptors on terminals of primary afferent neurons facilitate mechanical and thermal sensitization by lowering the activation threshold of second-order neurons and increasing the synaptic strength between nociceptors and spinal dorsal horn neurons (Seybold, 2009). These changes in intracellular signaling pathways contribute to long-term nociceptive neuron hyperexcitability resulting in persistent hyperalgesia. CGRP is widely distributed both peripherally and centrally, and is a known contributor to persistent musculoskeletal pain and neurogenic inflammation (Oku et al., 1987; Liu et al., 2011; Russo, 2015; Malon and Cao, 2016). Under normal physiological conditions, CGRP immunoreactivity labeling has been reported to be in approximately 40–46% of C-fiber, 33% of Aδ, and 17% of A α/β fiber neurons in lumbar DRG neurons (Lee et al., 1985; McCarthy and Lawson, 1990). However, following inflammation, persistent pain, neural injury, and/or limb immobilization, phenotypic changes are known to occur increasing the number of small as well as larger diameter DRG neurons showing CGRP immunoreactivity (Ohtori et al., 2001; Staton et al., 2007; Nishigami et al., 2009). Unlike smaller C and Aδ CGRP immunoreactive nociceptive fibers that terminate in the superficial laminae (I, II) of the dorsal horn, larger DRG fibers terminate in deeper spinal cord laminae that contain polymodal neurons which likely contributes to the development of persistent mechanical/thermal hyperalgesia or allodynia following musculoskeletal low back injury (Weng et al., 2003; Latremoliere and Woolf, 2009; Kimura et al., 2014).

The purpose of this initial study was to investigate whether passive SM could prevent the development of mechanical/thermal hyperalgesia, increase exploratory behavior, and lower spontaneous pain in a NGF-induced LBP model. In addition, this study served to determine whether SM potentially impacted NGF-mediated downstream CGRP changes in lumbar DRGs that likely contribute to LBP development. We used adult female rats in this initial SM study because there is growing evidence that women are more adversely affected by non-specific LBP than men (Schneider et al., 2006; Peterson et al., 2012; Coggon et al., 2017). Women also report greater LBP baseline intensity, more widespread LBP, more frequent recurrent LBP episodes, and experience more LBP-related disability compared to men (Hansson et al., 2006; Chenot et al., 2008; Peterson et al., 2012). Moreover, women utilize spinal manual therapy more often than men to help manage their musculoskeletal pain complaints (Hurwitz, 2012; Clarke et al., 2018). Ongoing NGF-induced LBP studies are investigating SM responses in adult male rats.

## Materials And Methods

**FIGURE 1:**
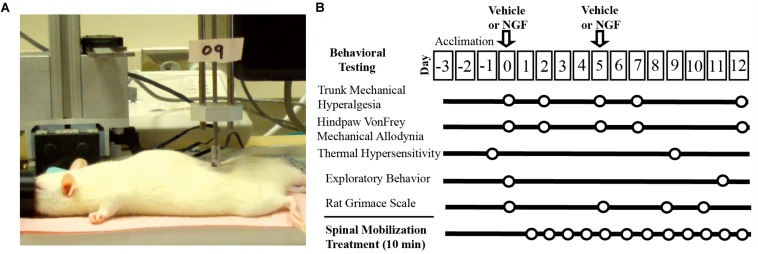
Experimental set-up and timeline. **(A)** Digital image of experimental set-up of L5 spinal mobilization delivered by a feedback control motor (0.9N, 1.2 Hz). **(B)** Experimental design and timeline.

All experiments were performed on adult female Sprague Dawley rats (187–270 g) and in compliance with the NIH Guide for the Care and Use of Laboratory Animals. All experiments were reviewed and approved by the University of Alabama at Birmingham Animal Care and Use Committee. Animals were group housed on a 12 h on/off light cycle with food and water provided *ad libitum*. For this initial study, no attempt was made to monitor or time stages of the estrous cycle due to the potential increases in stress-related responses associated with acquiring frequent vaginal smears. The experimental intervention, design and time course for our studies is displayed in Figure 1.

### NGF Intramuscular Injections

Injections of NGF were made into the left multifidus muscle at the vertebral level of L5 (3 mm lateral to the spinous process) on Days 0 and 5 under brief isoflurane anesthesia as previously described (Hoheisel et al., 2013). NGF solution (0.8 μM; human recombinant, Sigma-Aldridge, St. Louis, MO, United States) was injected in fifty microliters of vehicle (phosphate-buffered saline, PBS; pH 7.2–7.3). This concentration of NGF has been shown to induce hyperalgesia when intramuscularly injected in animals and humans (Deising et al., 2012; Hoheisel et al., 2013; Weinkauf et al., 2015). Similar lumbar muscle injections of vehicle (VEH [PBS, 50 μl]) served as a control.

### Treatment Groups

Animals (*n* = 32) were divided into 4 groups (VEH, NGF, VEH + SM, NGF + SM). Passive SM treatment began on Day 1 (the day following the first NGF or VEH injection) and was performed daily for 10 min using a computer controlled feedback motor (Figure 1) to deliver forces equivalent to 0.9N at 1.2 Hz under light isoflurane anesthesia (1–2%). For Day 5, SM treatment preceded the 2nd NGF injection to minimize any potential dispersal effect. To control for daily isoflurane exposure, all animal groups received 10 min of isoflurane daily under the same experimental conditions regardless of whether or not they received concurrent SM treatment. The laboratory personnel delivering SM was blinded to the injection content, but not to whether animals were to receive SM treatment. All behavioral testing was performed by the same individual in the same testing environment between 7 and 11:30 a.m.

### Measurement of Mechanical/Thermal Hyperalgesia, Exploration, and Spontaneous Pain

All animals were habituated to the testing environment and laboratory personnel beginning 2–3 days prior to onset of data collection and 30–60 min prior to designated testing. To test for trunk mechanical hyperalgesia, pain pressure threshold of the lumbar paraspinal muscle was tested bilaterally at L5 using a Bioseb SMALGO^®^ algometer with a blunt 5 mm tip which primarily stimulates deep tissue nociceptor response (Kosek et al., 1999). The head of the animals was briefly covered by a towel (calming effect) and mechanical pressure (g) was steadily applied with increasing intensity over the L5 paraspinal muscle until a pain-related reaction (withdrawal behavior, escape movements) was elicited (Hoheisel et al., 2013). Mechanical testing was performed 4x/experimental test day (Days 0, 2, 5, 7, 12) with a period of at least 5 min between consecutive trials. To determine if lumbar NGF injection caused distant mechanical allodynia, we examined hindpaw 50% withdrawal threshold response to mechanical stimulation using the von Frey (VF) “up and down method” (Dixon, 1980; Chaplan et al., 1994). Response thresholds were measured by calibrated VF filaments [Stoelting, Wood Dale, IL, ranging from 3.61 (0.407 g) to 5.46 (26 g) bending force] applied to the midplantar surface of the hindpaw (Dixon, 1980). Hindpaw mechanical testing was performed prior to the trunk algometry. Hindpaw thermal hyperalgesia testing using a hotplate assay (50°C; IITC Life Science, Woodlawn Hills, CA, United States) was performed on separate days than mechanical testing (Days –1, and 9) with a cut-off latency of 90s to prevent tissue injury.

The functional and spontaneous pain assays included the open field test and rat grimace scale (RGS). To prevent overstimulation of animals, these assays were typically performed on alternate days to reflexive pain behavioral assays (Figure 1). To test for alterations in exploratory activity during LBP, we performed automated open field exploratory activity including total movement distance (cm) and number of rearing events over 10 min duration using a Tru-Scan Activity Monitoring System (Colborne Instruments) on Days 0 and 11. To test spontaneous pain, we performed RGS scoring (Sotocinal et al., 2011). RGS testing was performed in the mornings between 7 and 9 a.m. and always prior to any mechanical or thermal trunk/hindpaw testing. To evaluate facial expressions consistent with grimacing to acute and severe pain states, rats were placed in clear plexiglass cubicles (20.3 × 10.2 cm) with video cameras at either end. Rats were video recorded for a period of 20 min and still-frame digital images taken every 2 min for RGS scoring by a blinded experimenter having no prior contact with the animals (Sotocinal et al., 2011). The RGS consisted of scoring a total of 4 action units: orbital tightening, nose bulge, ear position, and whisker changes as previously described (Sotocinal et al., 2011). Randomized images were assigned a value of 0, 1, or 2 for each of the four RGS action units: 0 was absent appearance of the unit, 1 was a moderate appearance of the unit, and 2 was an obvious appearance of the unit. RGS was performed on Days 0, 5 + 4 h, 8 and 10 (Figure 1).

### DRG CGRP-Immunofluorescence Microscopy

**FIGURE 2:**
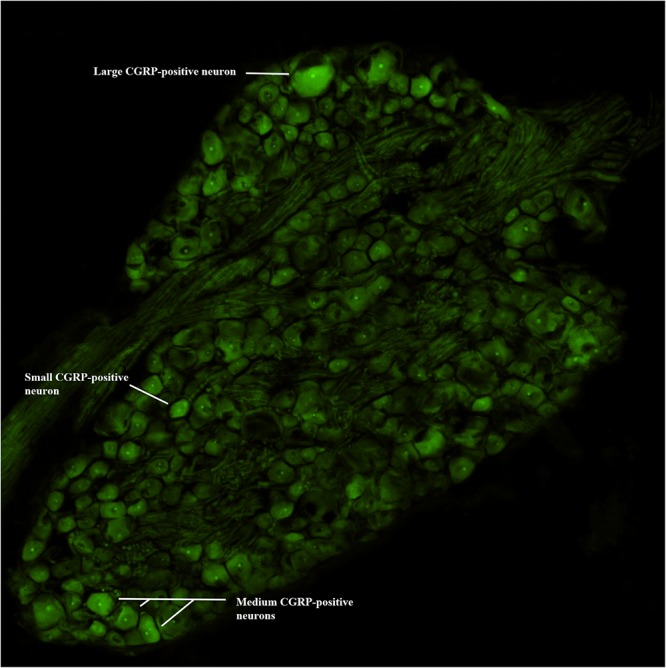
CGRP-immunofluorescence. Digital image of a lumbar DRG with examples of small, medium, and large CGRP-positive immunoreactive neurons.

Following Day 12 experimental testing, rats were euthanized and transcardially perfused with 4% paraformaldehyde in phosphate buffer (pH 7.4). Lumbar DRGs (L1–L6) were harvested bilaterally and fixed overnight at 4°C in 4% paraformaldehyde. Following fixation, DRGs were placed in 30% sucrose 1–2 days at 4°C, embedded in Tissue TEK OCT compound and quickly frozen until sectioned on a cryostat at 15 μm. Sections were washed in PBS with 0.3% Triton X-100 (Fluka) and 1% normal goat serum for 1 h, then incubated in primary antiserum, rabbit antiserum to CGRP (1:1000; Immnunostar, Hudson, WI, United States) overnight at 4°C. After 4 washes, sections were incubated in secondary antiserum, donkey anti-rabbit Cy-3 label IgG (1:200; Jackson Immunoresearch; West Grove, PA, United States) for 2 h. As a specificity control, certain slides were processed as described without primary antibody. Slides were coverslipped with Fluorogel II with DAPI (Electron Microscopy Sciences, Hatfield, PA, United States) and imaged under an epifluorescence microscope (Nikon Eclipse). Images were captured (10x) using a digital camera (Nikon) and analyzed using Nikon Elements^®^ (v4.2 software) by an individual blinded to the experimental groups. DRG sections having a large circumference and cell/nerve root ratio were evaluated bilaterally from each lumbar level. One section per segmental DRG level (from 3 animals) was analyzed and cells with identifiable nuclear profiles were outlined using Nikon artificial overlays to quantify the number of CGRP-positive labeled neurons and determine their cross-sectional area. Those values were normalized to the total number of DRG neurons with nuclear profiles to provide the percentage of CGRP-positive labeled neurons. Subpopulations of CGRP-positive neurons were also categorized based on cross-sectional area using the criteria set by Noguchi and colleagues (Fukuoka et al., 2001; Obata et al., 2003). Neurons were separated into small-sized (<600 μm^2^), medium-sized (600–1200 μm^2^) and large-size (<1200 μm^2^) cell profiles (Figure 2). A single DRG section from each segmental level from 3 animals were averaged for each experimental group, and expressed as a mean percentage (± standard deviation; SD).

### Statistical Analysis

The normality assumption was evaluated using Q-Q plots, and all data presented as ± SD. The effect of time was evaluated using repeated measures analysis of variance (ANOVA). Differences among experimental groups were determined by ANOVA followed by Tukey’s *post hoc* test as appropriate. A value of *p* < 0.05 was considered to be statistically significant. All the analysis was conducted using SAS 9.4 (Cary, NC).

## Results

### Mechanical and Thermal Hyperalgesia/Allodynia and SM

**FIGURE 3:**
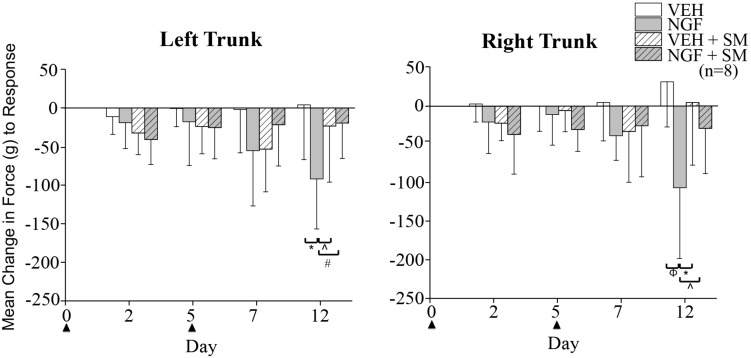
Trunk mechanical hyperalgesia. Mean change from baseline of magnitude of mechanical trunk stimulus required to elicit a pain response (escape, withdraw behavior) following left VEH (50 μl; phosphate-buffer solution/PBS) or NGF (50 μl, 0.8 μM) injections (▲) and daily spinal mobilization (10 min; ∼0.9N). Female rats developed bilateral mechanical hyperalgesia that was prevented by daily spinal mobilization. Statistical analyses were performed by ANOVA followed by Tukey’s test at each time point and data are presented as mean ± SD (*P = 0.006, ^∧^*P* = 0.04, ^#^*P* = 0.03, ^Φ^ *P* = 0.001).

**FIGURE 4:**
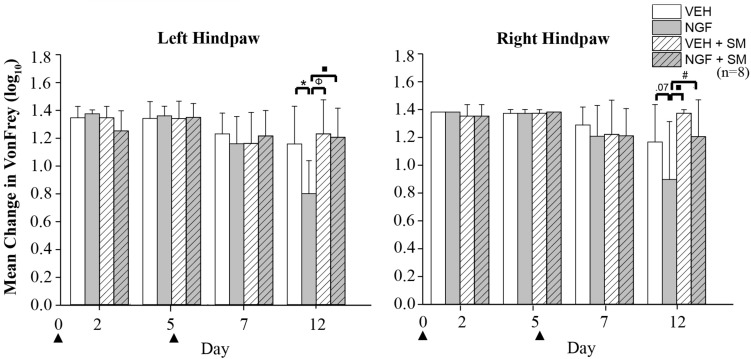
Hindpaw mechanical allodynia. Mean change in von Frey test of left and right hindpaw following two left injections (▲) of vehicle [phosphate-buffer solution (PBS); 50 μl] or nerve growth factor (NGF (50 μl, 0.8 μM) with and without spinal mobilization (SM). On Day 12 on the left hindpaw, significant differences were found between VEH and NGF (*P = 0.006), NGF and VEH + SM (^Φ^ *P* = 0.001) and NGF and NGF + SM (^■^*P* = 0.002); right hindpaw differences were found between NGF and VEH + SM (^■^*P* = 0.002) and NGF and NGF + SM ^(#^*P* = 0.03). Statistical analyses were performed by ANOVA followed by Tukey’s test at each time point and data are log transformed and reported as mean ± SD.

**FIGURE 5:**
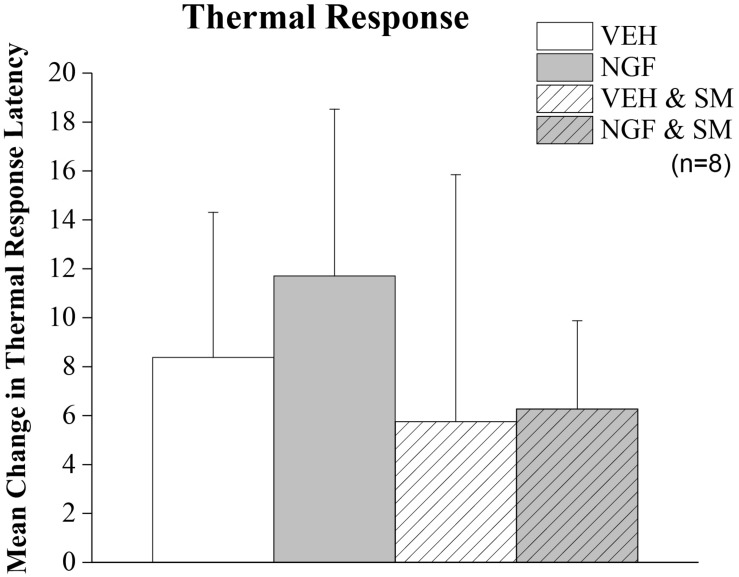
Thermal allodynia. Mean change in thermal hindpaw response latency to 50°C stimulus. Rats were tested on Day –1 and Day 9. Trunk injection of NGF did not significantly alter distant noxious hindpaw thermal response latencies. Statistical analyses were performed by ANOVA and data reported as mean ± SD.

By Day 12, significant trunk NGF group differences were demonstrated bilaterally when compared to the three other treatment groups (VEH [left *P* = 0.006, right *P* = 0.001]; VEH + SM [left *P* = 0.04, right *P* = 0.006); NGF + SM; [left *P* = 0.03; right side *P* = 0.04)] (Figure 3). Daily SM prevented Day 12 NGF-induced trunk mechanical hyperalgesia, maintaining the magnitude of change in mechanical trunk stimulus at or below Day 2 levels throughout experimental period (Figure 3). L5 SM by itself (VEH + SM) did not create trunk hyperalgesia to mechanical stimulation. NGF injections in female rats also induced the development of ipsilateral hindpaw mechanical allodynia by Day 12 which was effectively prevented by SM treatment (Figure 4). No significant changes in hindpaw thermal allodynia were noted between groups (Figure 5).

### SM and Exploratory Activity in LBP Animals

**FIGURE 6:**
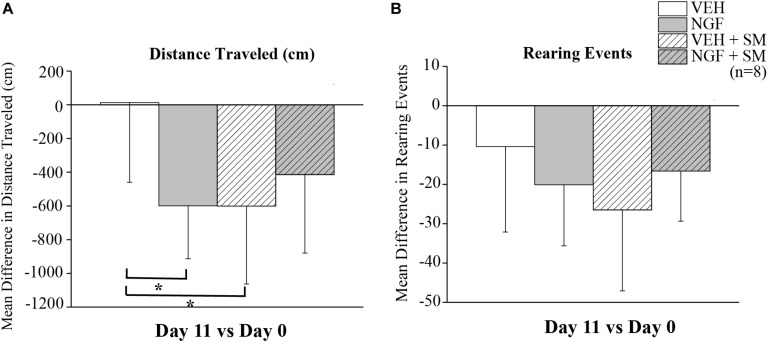
Exploratory behavior. Mean difference in exploratory distance traveled (cm) **(A)** and rearing events **(B)** on Day 11 compared to Day 0. NGF and VEH + SM animals traveled less total distance than VEH injected animals over 10 min of open field testing (*P = 0.04). There were no differences in total distance traveled between VEH and NGF + SM groups. In addition, no differences in rearing events were noted. Statistical analyses were performed by ANOVA followed by Tukey’s test and data reported as mean ± SD.

To assess the effect of NGF and SM treatment on rat exploratory behavior, mean differences in total distance traveled (cm) and rearing events over 10 min were measured on Day 0 (prior to 1st NGF injection) and Day 11 using a Tru-Scan Activity Monitoring System when LBP had fully developed. Compared to the VEH group, the NGF-injected and VEH + SM animals traveled significantly less total distance (*P* = 0.04, Figure 6A). Animals in the NGF + SM group had no differences in total distance traveled compared to the VEH group (Figure 6A). While the NGF + SM group exhibited more mean exploratory activity compared to the NGF group, this increase in total travel distance failed to reach significance (Figure 6A). For rearing events, all groups exhibited an overall decrease in the number of rearing events on Day 11 compared to baseline (Day 0) but no differences were noted between groups (Figure 6B).

### SM Prevents Spontaneous Pain in LBP Animals

**FIGURE 7:**
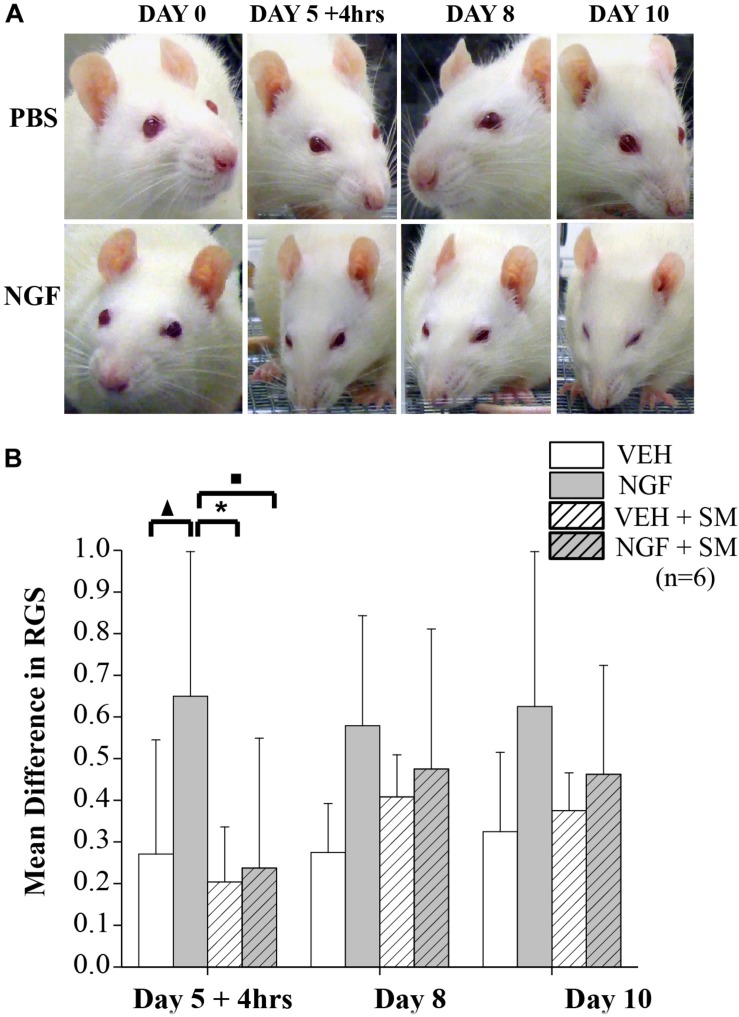
Rat grimace scale (RGS). **(A)** Representative images of facial grimacing observed due to NGF-induced low back pain. Four action units (orbital tightening, ear changes, cheek, and whisker change) are analyzed and scored. Note changes in spontaneous pain shown by orbital tightening and whisker retraction presented in the NGF group beginning as early as 4 h after the 2nd NGF injection delivered on Day 5. **(B)** Mean differences in RGS from Baseline on Days 5 + 4 h (4 h after 2nd NGF injection), 8 and 10. Compared to the VEH group the mean difference in RGS scores for the NGF group was significantly greater on Day 5 + 4 h (^▲^*P* = 0.03). Compared to the NGF group, the VEH + SM (*P = 0.01) and NGF + SM group (^■^*P* = 0.02) were significantly decreased. While the NGF group demonstrated higher mean RGS differences than other groups on Days 8 and 10, these changes did not reach statistical significance. Statistical analyses were performed by ANOVA followed by Tukey’s test and data reported as mean ± SD.

To assess spontaneous pain in NGF-injected animals and the potential impact of SM, we used RGS scoring as depicted in Figure 7A. Video recordings were performed on Days 0, *5+4* h, 8, and 10 with Day 5 recordings occurring 4 h after the 2nd NGF injection (Figure 1). Compared to baseline values, NGF increased RGS spontaneous pain scores on each day tested, however these increases reached significance only on Day 5 + 4 h (*P* = 0.03, Figure 7B). It should be noted that there were no significant differences in spontaneous pain scores between the VEH, VEH + SM and NGF + SM group at any time point and that VEH group RGS scoring remained fairly consistent throughout the experimental period (Figure 7B).

### SM Decreases CGRP-Positive DRG Neurons

**FIGURE 8:**
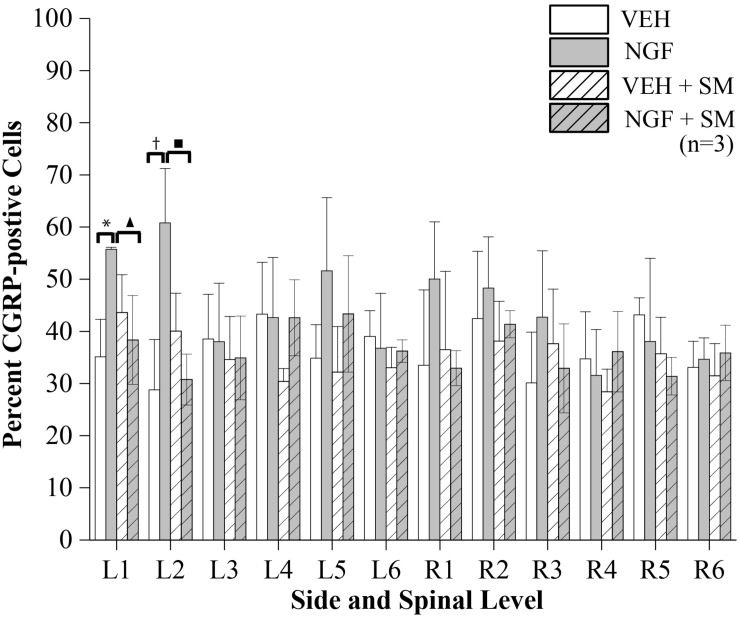
CGRP-positive Cells. The percent of CGRP-positive cells of lumbar DRG neurons having DAPI labeled nuclei. NGF injections (left side at L5) resulted in significant increases in CGRP-positive cell profiles at L1 and L2 on the left compared to the VEH and NGF + SM groups (*P = 0.02, ^▲^*P* = 0.05, ^†^*P* = 0.007, ^■^*P* = 0.01). Statistical analyses were performed by two-way ANOVA followed by Tukey’s test at each level and data reported as mean ± SD.

NGF injections into the left L5 multifidus muscle resulted in an increase in the mean percentage of CGRP-positive DRG neurons at the left L1 and L2 segmental levels in the NGF group compared to the VEH (*P* = 0.02) and NGF + SM (*P* = 0.05) groups (Figure 8). Despite an elevation, CGRP-positive cell percentage did not significantly change contralaterally at the R1 and R2 in the NGF group relative to the other groups (Figure 8). The majority of lumbar DRGs among the VEH group exhibited a range of 30–40% of CGRP-positive labeled cells, whereas the upper lumbar DRGs in the NGF group demonstrated 45–60% CGRP-positive labeled cells (Figure 8). The NGF + SM group demonstrated similar CGRP-positive cell percentages as the VEH group across the majority of lumbar DRG levels (Figure 8). Among the NGF animals, the majority of CGRP-positive cells (58.4%) were small neurons (<600 μm^2^), followed by 33.5% medium (600–1200 μm^2^) and 8.1% large (>1200 μm^2^).

## Discussion

In the current study we investigated whether passive SM prevents the development of local (trunk) mechanical hyperalgesia and distant (hindpaw) allodynia in a NGF-induced LBP model, as well as, if SM decreases the number of CGRP-positive lumbar DRG neurons as a potential mechanism of action for any pain-related behavioral outcomes. To our knowledge, this is the first preclinical study demonstrating the effects of SM on muscular LBP and testing its ability to improve functional activity, prevent local and distant somatosensory alterations, and alleviate spontaneous pain potentially through the suppression of pain-related neuropeptides in primary afferent neurons.

Previous studies using this NGF-induced model of LBP were limited to adult male rats and demonstrated the development of unilateral localized (trunk) mechanical hyperalgesia (Hoheisel et al., 2013; Zhang et al., 2017; de Azambuja et al., 2018). This effect began within 4 h after the second NGF injection and continued through Day 14. Our data were in general agreement as there was the presence of trunk hyperalgesia at Day 12. However, we found that unilateral NGF injections in female rats resulted in bilateral trunk mechanical hyperalgesia and unilateral hindpaw mechanical allodynia (Figures 3, 4). Sex-related differences in both clinical and experimentally induced pain models are well established and widely documented (Mogil and Bailey, 2010; Sorge et al., 2015; Sorge and Totsch, 2017). Evidence that this preclinical NGF-induced LPB model may have sex-related differences in LBP is important as this finding would only strengthen its potential translational ability. Similar studies investigating the effects of SM in NGF-induced LBP in adult male rats are ongoing. We did not find that NGF-induced LBP or SM altered hindpaw thermal allodynia, while increases in latency responses among all groups was attributed to increased familiarity with the behavioral testing environment.

Current approaches in pain research are attempting to improve the translation of animal models to the human condition. One way to accomplish this is to evaluate more functional pain assays that show depressed activity during pain states. Previous studies using the NGF model did not utilize functional assays. Here we found for the first time that in addition to somatosensory alterations detected by reflexive pain behavioral assays, the NGF LBP model also induces non-reflexive LBP behaviors. Low back NGF injections into the lumbar multifidus muscle decreased total distance traveled compared to the VEH group (Figure 6). The overall decline in exploratory behavior among all experimental groups on subsequent testing is common and can be attributed to previous exposure/increased familiarity equating to a decline in environmental novelty. Our finding that NGF-induced LBP impacts functional behavior is important as it provides opportunity to evaluate how both non-pharmacological and pharmacological treatments improve pain assays and functional activities. It also opens up the opportunity to determine the underlying pathophysiological mechanisms of reflexive and functional pain behaviors, which may help to inform the optimization of current LBP treatments and identify new therapeutic targets in the future.

In addition to exploratory behavior, we examined spontaneous pain for the first time in the NGF model using the RGS. We found that NGF trunk injections increased RGS scoring throughout experimental testing, with significance achieved only during the early development of the persistent phase of LBP after the 2nd NGF injection but scores remained elevated throughout the entire study. Notably, SM prevented an increase in spontaneous pain immediately following the 2nd injection evidenced by no differences being found between VEH, VEH + SM, and NGF + SM groups. Electrophysiology experiments using this same NGF LBP model have demonstrated that spinal cord dorsal horn latent sensitization from the 1st NGF injection is activated very quickly (within 1–2 h) following the 2nd NGF injection and manifests itself as increased neural resting activity and evoked response, increased convergent input, and the appearance of new receptive fields (Hoheisel et al., 2013; de Azambuja et al., 2018). The acute effects of NGF’s actions were enough to influence significant increases in spontaneous pain. Our finding is similar to initial studies wherein a rodent grimace scale was able to detect the acute onset of pain for hours but did not detect increases when the pain persisted for days or weeks (Langford et al., 2010; Sotocinal et al., 2011) unless the pain was very severe (Akintola et al., 2017). It was proposed that this greater sensitivity to acute changes with RGS may reflect that in chronic pain the facial grimace changes are less likely detected as there are environmental advantages to suppressing a painful face (Sotocinal et al., 2011). Importantly, based upon the acute timing of SM’s effects on RGS in the NGF model, SM may be acting in part to prevent the pathophysiological mechanisms leading to dorsal horn latent sensitization. NGF acts through its receptors to have effects on many downstream targets and can upregulate many pro-nociceptive substances (neuropeptides, etc.) including CGRP in primary afferent neurons that are essential to the development and maintenance of persistent pain (Jankowski and Koerber, 2010). As significant increases in the RGS were only found acutely and SM was able to suppress that phase, it suggests that the actions of daily SM prevent NGF-induced dorsal horn neuronal hyperexcitability, which then suppresses several pain mechanisms that lead to persistent LBP. This model using multiple NGF injections may also reflect how muscle injury and inflammation can lead to increases in NGF, which may act through mechanisms like nociceptive (hyperalgesic) priming to change the phenotypic expression of neurons and result in a greater likelihood of reoccurrence or transition from acute to chronic LBP (Joseph and Levine, 2010; Hoheisel et al., 2013; Hoheisel and Mense, 2015). That SM was able to prevent the onset of the persistent LBP phase in this model supports further mechanistic studies of LBP and the effects of SM in this model.

To begin exploring the underlying mechanisms of NGF-induced LBP and how SM may alter those mechanisms to suppress pain, we examined the NGF-dependent pain neuropeptide CGRP in lumbar DRGs. Sensitization of sensory neurons is considered an important step in the transition from acute to chronic pain and NGF has been linked to upregulation of the pain-related neuropeptide CGRP within the DRG (Malcangio et al., 1997; Supowit et al., 2001). CGRP upregulation in the DRGs has been reported in other animal LBP models such as intervertebral disc injury, disc degeneration, and inflammation (Lee et al., 2009; Miyagi et al., 2011, 2014). We found that NGF injections into the left multifidus muscle at L5 significantly increased CGRP-positive cell expression at L1 and L2 DRGs on the ipsilateral side (Figure 8). These findings of increased CGRP-positive neurons in the upper lumbar DRGs are in agreement with the segmental innervation of spinal tissues at the L5 vertebral level (where our paraspinal injections were performed). Lumbar spinal tissues have multi-segmental innervation with the greatest representation occurring in the upper lumbar segments. For example, distribution of DRG neurons labeled following application of the fluorescent neurotracer 1,1′dioctadecyl-3,3,3′3′-tetramethylindocarbocyanine (DiI) into muscular tissues at the level of the rat iliac crest (near the rat L5 spinous process) was found to be the greatest at L2 (49.5%) with 95.5% of DiI labeled DRG neurons being found at the combined L1 and L2 levels (Takahashi et al., 2003). Similarly in a separate study, 70% of labeled neurons resulting from DiI injected into the L4 left multifidus were found to be in located in the L2 and L3 DRGs (Ohtori et al., 2003). These studies support our findings that the greatest increases in CGRP-positive cells occur in the upper lumbar DRGs given the anatomical location of our NGF injections. Other deep lumbar structures such as the rat L5/6 disc (Ohtori et al., 1999) and L5/6 facet joint (Suseki et al., 1996) also demonstrate multi-segmental innervation by DRGs spanning from T13-L6 and L1-L5 respectively. Forty-seven percent of L1-L5 DRG neurons were reported to be CGRP-immunoreactive following an L5/6 disc injection, (Ohtori et al., 2002) while a somewhat lower percentage (38%) was reported following a similar L5/6 facet injection (Ohtori et al., 2000). These CGRP-related studies support earlier work showing that approximately 40% of DRG cells express CGRP immunoreactivity (Lee et al., 1985). In our work, we found similar percentages of lumbar DRG CGRP-positive neurons labeled among the vehicle group (Figure 8). NGF injections increased CGRP-positive percentages to above 50% of neurons primarily in the upper lumbar DRGs. This upper lumbar location also coincides with NGF enhanced extracellular neuronal activity from the L2 spinal dorsal horn using this same NGF-induced LBP model (Hoheisel et al., 2013). A key new finding of the current work is that lumbar SM effectively prevented and/or attenuated increases in CGRP-positive DRG sensory neurons following muscular NGF injections. The specific SM-related mechanisms responsible for CGRP (and perhaps other pain-related neuropeptides) normalization are not clear at the present but will be investigated in greater detail in both sexes in the future.

## Conclusion

Data from the current study show for the first time that passive SM has a preventative effect on the development of trunk mechanical hyperalgesia, decreases spontaneous pain, and attenuates CGRP-related response in lumbar DRG neurons caused by NGF-induced LBP in female rats. Unlike previous reports using male rats, female rats demonstrated bilateral trunk (local) mechanical hyperalgesia and ipsilateral hindpaw (distant) mechanical allodynia. As the understanding of sex-differences is an important consideration to manage the global problem of pain, this NGF model may allow mechanistic study of sex differences specific to muscular LBP. Early development of spontaneous pain was mitigated by daily short-duration SM treatment. Unilateral NGF injections into the L5 multifidus muscle increased CGRP immunoreactivity in primarily small DRG neurons located in the upper lumbar (L1 and L2) segments. This NGF-induced increase in CGRP-positive DRG neurons was also prevented and/or attenuated by mechanical stimulation related to passive SM. Much more work is needed to fully characterize the effects of SM in this NGF-induced LBP model, identify sex differences, and establish the biological mechanisms responsible for this therapeutic response to SM as well as to other non-pharmacological manual therapy interventions for LBP.

## Data Availability Statement

The datasets for this article are not publicly available because of security issues. Requests to access the datasets should be directed to WR, wreed@uab.edu.

## Ethics Statement

The experimental protocols for animal usage were reviewed and approved by UAB Institutional Animal Care and Use Committee following the National Institutes of Health guide for the care and use of laboratory animals (NIH Publications No. 96-01) revised in 1996.

## Author Contributions

WR, JL, TN, and RS conceptualized and designed the study. WR, JL, CL, RS, CY-F, TN, JG, DM, CH, ME, and PL contributed to acquisition, analysis, and interpretation of data. PL performed the statistical analysis. WR and JL wrote the first draft of the manuscript. All authors contributed to manuscript revision and approved the submitted version.

## Conflict of Interest

The authors declare that the research was conducted in the absence of any commercial or financial relationships that could be construed as a potential conflict of interest.
